# Chronic Condition and Risk Behaviours in Portuguese Adolescents

**DOI:** 10.5539/gjhs.v6n2p227

**Published:** 2014-02-20

**Authors:** Teresa Santos, Mafalda Ferreira, Maria Celeste Simões, Maria Céu Machado, Margarida Gaspar de Matos

**Affiliations:** 1 Faculdade de Motricidade Humana/Universidade de Lisboa (FMH/UL), Centro de Malária e Doenças Tropicais, Instituto de Medicina Tropical, Universidade Nova de Lisboa (CMDT/IHMT/UNL), Portuga; 2 Departamento de Pediatria do Hospital de Santa Maria, Portuga

**Keywords:** adolescence health, chronic condition, protective contexts, risk behaviours

## Abstract

Living with a chronic condition (CC) in adolescence has been historically considered protective for risk behaviours. However, research from the last decade suggest that when compared with healthy peers, adolescents living with a chronic condition can engage in risky behaviours in a similar if not higher rate than their counterparts living with out a CC. This study aims to characterize and evaluate the impact of 1) living with a chronic condition (CC), and 2) how the perception of living with a CC affects school participation, and its association with risk/protective behaviours (drunkenness, physical fight, sadness and self-harm). For this purpose 4 groups were identified: adolescents with mostly healthy behaviours, adolescents with mostly risk behaviours, adolescents with mostly risk-internalizing behaviours and adolescents with mostly risk-externalizing behaviours.

A large sample was included in this study, composed by 3494 Portuguese adolescents with an average age of 15 years, who participated in the Portuguese Survey of Health Behaviour in School-aged Children/WHO (HBSC).

Main results show that adolescents living with a CC have more risk-internalizing behaviours when compared to adolescents without CC, who present more healthy behaviors. Furthermore, adolescents that report that having a CC affects school participation show more risky behaviours than those not affected by a CC who present more healthy behaviours. Boys with a CC show more healthy behaviours, and those who feel that the CC affects school participation present more risky behaviours. On the other hand, girls with a CC have more risk-internalizing behaviours and less healthy behaviours

It is important to point out that dolescents living with a CC represent a vulnerable group, and may engage in experimental/risky behaviours as likely as their non CC peers. Thus, potential benefits can arise from reinforcing interventions within protective contexts (family/peers/school setting). Health/education professionals, more than considering risk behaviours as dangerous in themselves, should offer adolescents with a CC an opportunity to reflect on their own decisions. Educational programs would benefit from looking at risk behaviors more from an experimentation perspective, focusing on constructive ways to help adolescents with CC to proceed into adulthood in a more appropriate developmental way.

## 1. Introduction

Adolescence comprises a period of profound biopsychosocial changes (Sprinthall & Collins, 1999) with adolescents facing potentially difficult challenges. This period of life may become even more challenged in the presence of a chronic condition and its implications (Michaud, Suris, & Viner, 2007; Simões, Matos, Ferreira, & Tomé, 2010). Diseases’s adaptation process is not homogeneous and can be quite variable depending on various specific individual/contextual factors, and also on the type of condition and emerging limitations, being even worse in the presence of both (Barros, 2009; Lee et al., 2013; Sawyer et al., 2002). Chronic condition relates to any extended and irreversible illness and condition such as asthma, congenital heart disease, epilepsy and diabetes, which are the most prevalent in adolescence (Barros, 2009).

Data indicates that adolescents with disabilities are less exposed to protective factors compared to healthy peers (Blum, Kelly, & Ireland, 2001; Svetaz, Ireland, & Blum, 2000). Protective factors refer to conditions that improve people’s resistance to risk factors and disorders (Coie et al., 1993), which can be, for adolescents with a CC, the condition or the child itself, family, community, or relationships with peers (Nylander, Seidel & Tindberg, 2013).

Adolescent development involves experimental behaviors that can be understood as a normal part of teenage development (Steinberg, 2004). However, sometimes it can lead to risky behaviours that compromise health, quality of life, or life itself (Jessor, 1991). Early onset of sexual activities, unsafe sexual practices, substance use or abuse and violent or antisocial behaviours are all examples of behaviours that might put an adolescent’s health at risk. Therefore, risky behaviour can be defined as the participation in activities that may compromise adolescents’ physical and mental health, mainly initiated due to developmental characteristics, or influenced by the surrounding environment (i.e. peers and family) (Steinberg, 2004). In fact, a Canadian study showed that, currently, there is a substancial number of young people that consume alcohol, tobacco and drugs and that the simultaneous use of these substances is also very widespread among users. Alcohol was the most prevalent used substance and it was rare to find only tobacco or drugs users (without the simultaneous consumption of alcohol), and, by grade 12, the majority of students were frequent users of alcohol, tobacco or drugs (Leatherdale & Burkhalter, 2012). However, if not early identified, risky behaviour can lead to significant individual, family and social issues (Steinberg, 2004).

In adolescents with chronic conditions, these exploring behaviours part of teenage development have historically assumed a protective role, helping to restrict opportunities that lead to risky behaviours (Frey, Guthrie, Loveland-Cherry, Park & Foster, 1997). Nevertheless, further investigations changed this perspective suggesting that adolescents with chronic conditions are doubly disadvantaged. Adolescents with a CC engage in risky behaviours in a similar if not higher rate, than healthy peers, and are more vulnerable to the adverse health outcomes that result from these behaviours (Blum et al., 2001; Kakleas, Kandyla, Karayianni & Karavanaki, 2009; Saunders, 2011; Sawyer, Drew, Yeo & Britto, 2007; Scaramuzza et al., 2010; Suris & Parera, 2005). Increasing data indicate that adolescents with chronic conditions are encompassed by fewer protective factors (Blum et al., 2001; Svetaz et al., 2000), engage in an equal or even higher amount of risk behaviours than their healthy counterparts (Blum et al., 2001; Sawyer et al., 2007; Suris, Michaud, Akre, & Sawyer, 2008; Suris & Parera, 2005) and there is evidence that health risk behaviours tend to cluster together (Suris et al., 2008; Brenner, & Collins, 1998; DuRant, Smith, Kreiter, & Krowchuk, 1999; Rhee, Yun, & Khang, 2007).

Thus, risky behaviour has a negative impact on adolescents’s general health and the consequences may be worse for vulnerable individuals, such as adolescents with a chronic condition.

Literature has showed that tobacco consumption is as common in young people with asthma and diabetes as in healthy peers (Precht, Keiding, & Madsen, 2003). Alcohol is thought to be the most frequently used substance by adolescents with chronic conditions, with little variation by diagnosis. Both girls and boys with CC seem to report fewer protective factors and more risk behaviours than their healthy peers. Some gender differences were also reported: girls with CC encompassed less individual protective factors, while boys with CC tend to over-report all indivdual risk behaviours, compared to healthy peers (Nylander et al., 2013). On the other hand, comsumption rates for other substances (eg, marijuana) and delinquent behaviour seem to be lower in young people with chronic conditions than in comparison groups (Britto, Garrett, Dugliss et al., 1998). It is important to recall that adolescents’s individual variables may predict and influence drinking patterns in adulthood (Windle & Windle, 2012), the specific period where chronic conditions can have a more effective protective role in alcohol consumption (Fat & Shelton, 2012; Liang & Chikritzhs, 2010; Kuntsche et al., 2009). Chronic conditions can also be associated with a slight elevated risk for self-harm (Barnes, Eisenberg, & Resnick, 2010). Issues related to sexuality and sexual identity are crucial due to the limitation of contact between peers and adolescents living with a chronic condition which may, as a result, interfere with opportunities for normal sexual experimentation and social development (Suris, Resnick, Cassuto, & Blum, 1996).

Still, findings about substance use among adolescents with chronic conditions are inconsistent and unclear (Sawyer et al., 2007; Geist, 2003). Therefore, risk behavioural factors and protective factors require further analysis (Filho & Ferreira-Borges, 2008; Bachman et al., 2008; Nylander et al., 2013).

The effects of a chronic condition extend beyond risky behaviours *per se* and can also compromise a healthy psychological development, cognitive skills, relationship with family/school/peers and health-related behaviours (Michaud et al., 2007). This way, adolescents living with a chronic condition can be at higher risk for a healthy emotional, behavioural and psychological development (Verhoof, Maurice-Stam, Heymans & Grootenhuis, 2012; Bernstein, Sore, Stockwell, Rosenthal, & Gallagher, 2011). They can also experience more adjustment problems (Oeseburg, Jansen, Groothoff, Dijkstra, & Reijneveld, 2010; Geist, 2003), as well as internalize symptoms such as depression (Vanhalst et al., 2013; Miyazaki, Amaral & Grecca, 2006), anxiety and social withdrawal, and externalize others through specific behaviors (Lavigue & Fier-Routman, 1992). Boys seem to be significantly more likely than girls to display behavioral and adjustment problems (Gortmaker, Walker, Weitzman, & Sobol, 1990), and girls show higher levels of anxiety (Matos et al., 2012), depression (La Greca, Swales, Klemp, Madigan, & Skyler, 1995), emotional distress (sadness, expressing depressive symptomatology) and suicide ideation (Suris, Parera, & Puig, 1996).

There is a large body of evidence linking policy and environmental change to desired behaviour (Ory, Jordan, & Bazzare, 2002) and schools seem to offer an ideal setting where policy’s and curricula can influence students (Kuntsche & Jordan, 2006; Mercer et al., 2003) and reduce risk factors for chronic conditions (O’Brien et al., 2010). It is suggested that, providing adolescents adequate information focused on the potential harm of simultaneous use of alcohol, tobacco and drugs may be a useful prevention strategy (Brière, Fallu, Descheneaux, & Janosz, 2011; Leatherdale & Burkhalter, 2012). Professionals should also provide stronger protection for these adolescents, to prevent risky behavior (Nylander et al., 2013).

In Portugal, HBSC study (Matos & Equipa Aventura Social, 2010, 2006) and Kidscreen study (Gaspar & Matos, 2008) indicate greater vulnerability of this population and the need for strengthening the main support structures (family and school), warning health and education systems for the global aspects of mental health, scholar and social integration and prevention of risk behaviours (Barros, 2009).

It seems therefore relevant to develop the following study aiming to characterize and evaluate the impact of: 1) having a chronic condition (CC), and 2) how CC affects school participation, in Portuguese adolescents; and its association with health and well-being related risk/protection behaviours, such as drunkenness, physical fight in the last year, sadness and self-harm.

## 2. Method

### 2.1 Participants

Based upon the data from the Portuguese study of 2010 HBSC, the sample of this study is composed by 3494 Portuguese adolescents (53.6% girls and 46.4% boys), with a mean age of 15 years (*SD*=1.29; *Median*=15,17) attending the 8^th^ and 10^th^ grades, randomly selected from 256 classes in 125 Portuguese national public schools.

### 2.2 Research Design and Questionnaire

A self-administered questionnaire from the Portuguese sample of the Health Behaviour in School-aged Children (HBSC) was used. Portugal was included as a full partner for the first time in 1996 and, since then, the study is carried on every 4 years. HBSC is a cross-sectional design study, a school-based, self-report questionnaire developed cooperatively between international researchers according to protocol. It is used in collaboration with the World Health Organization to assess children and adolescents’ mental and physical health (Currie et al., 2012). The aim of the study is to achieve a new and expanded understanding of health, health behaviour and well-being among adolescents within their social context, through the gathering of data that allows national and international comparisons (Roberts et al., 2007). Specially designed to be appropriate for adolescents aged 11-15, this survey consists of 75 items that measure background factors (e.g., socioeconomic status, family structure), individual and social resources (e.g., body image, school environment), health behaviours (e.g., smoking, dieting, sexual behaviour, violence), and health outcomes (e.g., life satisfaction, psychological well-being, and self-reported health).

### 2.3 Procedure

The national HBSC study (http://www.hbsc.org/) was approved by a scientific committee and the national ethics committee. The study is essentially descriptive and cross-sectional, correlational in nature.

This sample data was collected in 2010 and the 139 schools were randomly selected from the official national list of public schools and stratified by region. According to the international protocol, in each school, the class was the analysis’s unity and classes were randomly selected in order to meet the required number of students for each grade (Currie et al., 2012). The study followed all the research rules defined by the Portuguese Ministry of Education and Regional Offices of Education. All participating schools made available informed parental consent, required by the parent association of each school.

Questionnaires were sent to schools and, according to the protocol, teachers administered the questionnaires in the classroom with voluntary student participation. Confidentiality was ensured through anonymous responses to the questionnaire. Regarding the work on computing and data analysis, there was a restricted access to HBSC research team members. Details about the study procedures can be consulted in the national Portuguese reports (Matos & Equipa Aventura Social, 2006, 2010).

### 2.4 Measures

The international standard questionnaire consists of three levels of questions which are used to create national survey instruments: core questions that each country is required to include to create the international dataset; optional packages of questions on specific topic areas from which countries can choose from; and country-specific questions related to issues of national importance.

Survey questions cover a range of health indicators and health-related behaviours as well as the life circumstances of young people. Questions are subjected to validation studies and piloting at national and international levels (Roberts et al., 2009). For the purpose of this specific study, the questionnaire includes questions about risk/protective behaviours and chronic condition, and the used questions are presented in table 1.

**Table 1:** Questions about risk behaviours and chronic condition used in the present study

	Items	*Responses*	
Drunkenness	In the last 30th days, have you ever had so much alcohol that you were really drunk?	*1.*	*Never*
		*2.*	*1-2 times*
		*3.*	*3-5 times*
		*4.*	*6-9 times*
		*5.*	*10-19 times*
		*6.*	*20-39 times*
		*7.*	*More than 40 times*
Physical fight	During the past 12 months, how many times were you in a physical fight?	*1.*	*None*
		*2.*	*1 time*
		*3.*	*2 times*
		*4.*	*3 times*
		*5.*	*4 times or more*
Sadness	I’m so sad that it seems that I cannot stand it	*1.*	*Never or almost never*
		*2.*	*Sometimes*
		*3.*	*Often*
Self-harm	During the last 12 months, have you ever hurt yourself on purpose?	*1.*	*Never*
		*2.*	*1 time*
		*3.*	*2 times*
		*4.*	*3 times*
		*5.*	*4 times or more*
Chronic condition (CC)	Do you have any long term illness, incapacity, disability or health problem that you have been diagnosed by a doctor?	*1.*	*Yes*
		*2.*	*No*
*Participation in school*	*This prolonged illness, disability, health problem or disability affects your attendance and participation in school?*	*1.*	*Yes*
		*2.*	*No*

### 2.5 Data Analysis

Questionnaires were scanned and data entry was done using the Program Eyes and Hands forms, version 5.

The data was then entered into a database and analysed using the Statistical Package for Social Sciences, version 19.0 for Windows.

Descriptive statistics including frequencies, means and standard deviations were performed to give general descriptions of the data, using the Chi-square (χ^2^) tests to analyse differences between having or not a chronic condition. The level for statistical significance was set at p< .05. Only significant results were discussed.

The variables about risk behaviours, namely Drunkenness, Physical fight, Sadness and Self-harm were standardized through Z-score, and an analysis through K-cluster was performed, in order to assemble adolescents in groups by means of a K-cluster method.

## 3. Results

The majority of students in the sample do not have a chronic condition (80.4%) and from those who say they have a chronic condition (n=643), about one-fifth report that the chronic health condition affects their participation and attendance in school (18.7%). Analyzing the differences between gender and age group we can verify there are no differences between groups, in both cases (table 2).

**Table 2:** Pupils distribution according to gender/grade and chronic condition

	Gender						*Grade*					
	Boys		Girls				8^th^		10^th^			
	N	%	N	%	χ^2^	Df	N	%	N	%	χ^2^	*Df*
Chronic condition (N=3278)					.072	1					.292	*1*
No	1235	81.7	1400	79.2			1200	81.2	1435	79.7		
*Yes*	*276*	*18.3*	*367*	*20.8*			*278*	*18.8*	*365*	*20.3*		

**p≤ .001; **p≤ .01; *p≤ .05

Four groups were named according to the adolescent’s personal more frequent situation regarding drunkness, physical fights, sadness and self-harm (table 3): adolescents that, in general, present all risk behaviours were named “Adolescents with mostly risk behaviours” (group 1) (N=261, 8.3%); adolescents that only present risk-internalizing behavious such as feeling sad, were named “Adolescents with risk-internalizing behaviours” (group 2) (N=1354, 43%); adolescents that do not present such risk behaviours, were named “Adolescents with mostly healthy behaviours” (group 3) (N=1250, 39.7%); and adolescents that mostly present risk-externalizing behaviours such as excessive drinking, fighting and self harming were named “Adolescents with risk-externalizing behaviours” (group 4) (N=286, 9.1%). As seen in Table 3, a positive association means that a behavior/situation was found to be significantly more frequent in a specific group, whereas a negative association means that a behavior/situation was found to be significantly less frequent in a specific group (Maroco, 2007).

**Table 3:** Final Cluster Centers after a K-cluster analysis using four risk behavior variables (Drunkenness, Physical fight, Sadness and Self-harm) using standardized through Z-score

	Mostly Risk Behaviours (Group 1)	Internalizing Behaviours (Group 2)	Mostly Healthy Behaviours (Group 3)	Externalizing Behaviours (Group 4)
Drunkenness	.30	-.10	-.19	1.00
Physical fight	.39	-.30	-.30	2.38
Sadness	.73	.84	-1.00	-.15
Self-harm	3.04	-.24	-.31	-.21

The results of the chi-square test showed that adolescents with chronic condition have more internalizing behaviours (χ^2^=24.01(3), *p*≤ .001, 47.6%), while adolescents without chronic condition are more frequently in the cluster with mostly healthy behaviours (χ^2^=24.01(3), *p≤* .001, 41.3%). If the chronic condition affects their participation and attendance in school, the adolescents are more frequently in the cluster “mostly risk behaviours” (χ^2^=20.27(3), *p≤* .001, 20.3%), while adolescents who refer that chronic condition doesn’t affect their school participation and attendance are more frequently in the group with mostly healthy behaviours (χ^2^=20.27(3), *p*≤ .001, 37.2%).

Comparing the same variables, having a chronic condition diagnosed by a doctor and if that chronic condition affects attendance and participation at school, by gender, it’s possible to verify that boys with chronic condition are more frequently in the cluster “mostly healthy behaviours” (χ^2^=17.88(3), *p*≤ .001, 48.9%) while boys without chronic condition are more frequently in the cluster with “mostly risk behaviours” (χ^2^=17.88(3), *p*≤ .001, 12.7%). In the same way, boys whose attendance and participation in school were affected were more frequently in the cluster with “mostly risk behaviours” (χ^2^=16.54(3), *p*≤ .001, 26.3%). On the other hand, boys whose participation was not affected were more frequently included in the cluster “mostly healthy behaviours” (χ^2^=16.54(3), *p*≤ .001, 43.3%). Girls with chronic condition were found more frequently in the cluster “internalizing behaviours” (χ^2^=8.83(3), *p*≤ .05, 58.2%) and girls without chronic condition more frequently in the cluster “mostly health behaviours” (χ^2^=8.83(3), *p*≤ .05, 34.8%). If the chronic condition affects attendance and participation in school, no statistical significant differences were found for girls (Table 4).

**Table 4:** Cluster membrership distribution by CC and CC affecting school participation – total and by gender

	Mostly Risk Behaviours (Group 1)		Internalizing Behaviours (Group 2)		Mostly Healthy Behaviours (Group 3)		Externalizing Behaviours (Group 4)		Total	χ^2^	*df*
	N	%	N	%	N	%	N	%
Chronic condition (CC)										24.01***	*3*
Yes	70	11.6	288	47.6	199	32.9	48	7.9	605		
No	184	7.4	1046	41.9	1031	41.3	235	9.4	2496		
CC Affecting school										20.27***	*3*
Yes	25	20.3	57	46.3	26	21.1	15	12.2	123		
No	70	9.4	327	44.0	277	37.2	70	9.4	744		
Chronic Condition by Gender											
*Boy*										17.88***	*3*
Yes	69	6.0	343	29.8	563	48.9	177	15.4	1152		
No	32	12.7	82	32.7	98	39.0	39	15.5	251		
*Girl*										8.83*	*3*
Yes	38	10.7	206	58.2	101	28.5	9	2.5	354		
No	115	8.6	703	52.3	468	34.8	58	4.3	1344		
CC Affecting school Participation by Gender											
*Boy*										16.54***	*3*
Yes	15	26.3	15	26.3	14	24.6	13	22.8	57		
No	34	9.9	105	30.5	149	43.3	56	16.3	344		
*Girl*										*6.47(n.s.)*	*3*
Yes	10	15.2	42	63.6	12	18.2	2	3.0	66		
*No*	*36*	*9.0*	*222*	*55.5*	*128*	*32.0*	*14*	*3.5*	*400*		

* p< .05;

** p< .01;

*** p< .001

In bold – values that correspond to an adjusted residual ≥ │1.9│

## 4. Discussion

In the present study we sought to establish a relation between: 1) having a chronic condition (CC), and 2) how that condition affects/does not affect participation in school; and its association with health and well-being related risk/protection behaviours, such as drunkenness, physical fight in the last year, sadness and self-harm, in a sample of Portuguese adolescents.

Results showed that adolescents living with a chronic condition have more risk-internalizing behaviours, while adolescents without a chronic condition present more healthy behaviours. Adolescents living with a chronic condition and feeling that the CC affects their participation and attendance in school have more risky behaviours, while adolescents who refer that the chronic condition doesn’t affect school have more healthy behaviours. This data supports the hypothesis evidenced in the literature, proposing that adolescents with a chronic condition are doubly disadvantaged and more vulnerable, engaging in risky behaviours in a similar if not higher rate, when compared with healthy peers (Blum et al., 2001; Kakleas et al., 2009; Nylander et al., 2013; Sawyer et al., 2007; Saunders, 2011; Scaramuzza et al.., 2010; Suris & Parera, 2005). It is also demonstrated that responses to disease can be worst when 2 limitations occur (having a CC and feeling it affects school participation) (Lee et al., 2013; Barros, 2009; Sawyer et al., 2002). Comparing the same variables by gender, boys with a chronic condition report more healthy behaviours and boys without a chronic condition report more risky behaviours. Also boys whose attendance and participation in school was affected reported more risky behaviours. Boys whose participation was not affected reported more healthy behaviours. Girls living with a chronic condition present more internalizing behaviours and girls without a chronic condition more healthy behaviours. Also, this data supports the idea that girls have more internalized behaviours (Matos et al., 2012; Suris et al., 1996; La Greca et al., 1995) than boys.

Adolescents explore and experiment different life styles and typical adult behaviours for various reasons, mainly to promote independence from parents and develop an individual identity. Therefore, experimenting with disease management is an example of explorative behavior; it serves developmental needs and, in this way, is not considered the same as risk-taking. However, young people are not truly able to make rational decisions until the maturation of the brain (around the age of 23–25). Therefore, in order to promote a healthier adolescent development it is crucial to understand protective contexts, such as family, peers and school, and place human free choice as a central topic of discussion. This is where health professionals and educators should offer adolescents an opportunity to think and talk about their choices. More than considering risk behaviour as dangerous in itself, it is important to keep a dialogue with teens and promote education concerning risky behaviour to help them proceed toward adulthood in the safest way possible (Scaramuzza et al., 2010).

Both family and school constitute target groups for interventions that aim at promoting resilience and thus preventing health risk behaviours (Nylander et al., 2013). The school context (peer group) and social environment in which the adolescent is integrated has been identified as the most consistent predictor of substance use in adolescence (Kuntsche & Jordan, 2006; Kuntsche et al, 2009), highlighting the need to explore the behaviours associated with these specific contexts. In addition, motivation and perception of belonging to a culture or group are also other consistent predictors of substance use. Thus, these are crucial factors that require further analysis in order to enhance prevention of behaviours that are harmful to one’s health (Filho & Ferreira-Borges, 2008; Bachman, et al, 2008). Schools seem to offer an ideal setting where policies and curricula can influence students (Mercer et al., 2003) and help reduce risk factors for chronic disease, simultaneously (physical activity, dietary behaviour, and tobacco use) (O’Brien et al., 2010). Schools can help reduce risk behaviours in adolescents with chronic conditions, as in healthy adolescents, through the promotion of healthy lifestyles and the prevention of chronic conditions in future adulthood.

Healthcare professionals are also important both in primary and secondary prevention, assuming a relevant role in disseminating knowledge among parents, school staff and significant adults in young people’s leisure activities. Therefore, they should aim at strengthening protective factors helping the adolescent in order to ensure resilience and prevent health risk behaviour.

Two limitations must be addressed regarding this study. The first concerns the self-report nature of the surveys: self-reported data depends on selective memory and therefore the findings may be biased. The second limitation has to do with the extent to which the findings can be generalized. Though the number of cases allows it to be a nationwide study, the findings cannot be generalized beyond Portugal because the majority of the sample is Portuguese and there are specific cultural values and beliefs related to the variables being studied.

## 5. Conclusion

In addition to understanding the whole context in which adolescents are involved, it is important to rethink strategies. As the HBSC is a research and monitoring study, resulting in the construction of a coherent set of indicators that, taken together, provide a valid representation of health and lifestyles of adolescents, it also aims to inform/impact health education policies and national/international programs and interventions for adolescents, in order to promote health and prevent risk behaviours

## 6. Key-Findings

Portuguese adolescents with a CC can be a vulnerable group and engage in risky behaviours as likely as their non CC peers. Thus, potential benefits can arise from reinforcing interventions in protective contexts, such as family, peers and school.
